# Prognostic role of the systemic immune–inflammation index in upper tract urothelial carcinoma treated with radical nephroureterectomy: results from a large multicenter international collaboration

**DOI:** 10.1007/s00262-021-02884-w

**Published:** 2021-02-16

**Authors:** Keiichiro Mori, Irene Resch, Noriyoshi Miura, Ekaterina Laukhtina, Victor M. Schuettfort, Benjamin Pradere, Satoshi Katayama, David D’Andrea, Mehdi Kardoust Parizi, Mohammad Abufaraj, Wataru Fukuokaya, Claudia Collà Ruvolo, Stefano Luzzago, Sophie Knipper, Carlotta Palumbo, Pierre I. Karakiewicz, Alberto Briganti, Dmitry V. Enikeev, Morgan Rouprêt, Vitaly Margulis, Shin Egawa, Shahrokh F. Shariat

**Affiliations:** 1 Department of Urology, Medical University of Vienna, Währinger Gürtel 18-20, 1090, Vienna, Austria; 2 Department of Urology, The Jikei University School of Medicine, Tokyo, Japan; 3 Department of Urology, Ehime University Graduate School of Medicine, Ehime, Japan; 4 Institute for Urology and Reproductive Health, Sechenov University, Moscow, Russia; 5 Department of Urology, University Medical Center Hamburg-Eppendorf, Hamburg, Germany; 6 Department of Urology, CHRU Tours, Université François Rabelais de Tours, PRES Centre Val de Loire, Tours, France; 7 Department of Urology, Dentistry and Pharmaceutical Sciences, Okayama University Graduate School of Medicine, Okayama, Japan; 8 Department of Urology, Shariati Hospital, Tehran University of Medical Sciences, Tehran, Iran; 9 Research Division of Urology, Department of Special Surgery, The University of Jordan, Amman, Jordan; 10 Cancer Prognostics and Health Outcomes Unit, University of Montreal Health Centre, Montreal, Canada; 11 Department of Urology, University of Naples Federico II, Naples, Italy; 12 Department of Urology, IRCCS, European Institute of Oncology, Milan, Italy; 13 Martini-Klinik Prostate Cancer Center, University Hospital Hamburg-Eppendorf, Hamburg, Germany; 14 Department of Medical and Surgical Specialties, Radiological Science, and Public Health, Urology Unit, ASST Spedali Civili of Brescia, University of Brescia, Brescia, Italy; 15 Department of Urology, Vita Salute San Raffaele University, Milan, Italy; 16 Urology department, Sorbonne Université, ONCOTYPE-URO, AP-HP, Hôpital Pitié-Salpêtrière, GRC n°5, 75013 Paris, France; 17 Department of Urology, University of Texas Southwestern Medical Center, Dallas, TX USA; 18 Department of Urology, Second Faculty of Medicine, Charles University, Prague, Czech Republic; 19 Department of Urology, Weill Cornell Medical College, New York, NY USA; 20 Karl Landsteiner Institute of Urology and Andrology, Vienna, Austria; 21 European Association of Urology Research Foundation, Arnhem, Netherlands

**Keywords:** Upper tract urothelial carcinoma, Systemic immune–inflammation index, Nephroureterectomy

## Abstract

**Purpose:**

To investigate the prognostic role of the preoperative systemic immune–inflammation index (SII) in patients with upper tract urothelial carcinoma (UTUC) treated with radical nephroureterectomy (RNU).

**Materials and methods:**

We retrospectively analyzed our multi-institutional database to identify 2492 patients. SII was calculated as platelet count × neutrophil/lymphocyte count and evaluated at a cutoff of 485. Logistic regression analyses were performed to investigate the association of SII with muscle-invasive and non-organ-confined (NOC) disease. Cox regression analyses were performed to investigate the association of SII with recurrence-free, cancer-specific, and overall survival (RFS/CSS/OS).

**Results:**

Overall, 986 (41.6%) patients had an SII > 485. On univariable logistic regression analyses, SII > 485 was associated with a higher risk of muscle-invasive (*P* = 0.004) and NOC (*P* = 0.03) disease at RNU. On multivariable logistic regression, SII remained independently associated with muscle-invasive disease (*P* = 0.01). On univariable Cox regression analyses, SII > 485 was associated with shorter RFS (*P* = 0.002), CSS (*P* = 0.002) and OS (*P* = 0.004). On multivariable Cox regression analyses SII remained independently associated with survival outcomes (all *P* < 0.05). Addition of SII to the multivariable models improved their discrimination of the models for predicting muscle-invasive disease (*P* = 0.02). However, all area under the curve and C-indexes increased by < 0.02 and it did not improve net benefit on decision curve analysis.

**Conclusions:**

Preoperative altered SII is significantly associated with higher pathologic stages and worse survival outcomes in patients treated with RNU for UTUC. However, the SII appears to have relatively limited incremental additive value in clinical use. Further study of SII in prognosticating UTUC is warranted before routine use in clinical algorithms.

**Supplementary Information:**

The online version of this article (10.1007/s00262-021-02884-w) contains supplementary material, which is available to authorized users.

## Introduction

Upper tract urothelial carcinoma (UTUC) is a relatively rare malignancy, accounting for only 5%–10% of all urothelial carcinomas [1, 2]. To improve the oncological outcomes in patients with UTUC, preoperative predictive models have been developed to guide clinical decision-making and patient counseling [3–5]. These models are based on multifocality, tumor size, grade on biopsy and cytology, hydronephrosis, and imaging findings with the aim of stratification of tumors into low risk and high risk [1]; they are also useful for decision-making regarding treatment in conjunction with kidney-sparing procedures versus radical nephroureterectomy (RNU) with or without lymphadenectomy and perioperative chemotherapy. However, it is difficult to predict pathological features preoperatively because of the limited amount of tissue in biopsy specimens [6]. Moreover, despite modern imaging technologies, such as computed tomography (CT) and magnetic resonance imaging, it remains difficult to achieve sufficient prognostic accuracy to facilitate formulation of individualized treatment strategies for patients with UTUC [1, 7]. Reliable preoperative prognostic factors are of particular interest in this regard, given that patients at high risk of tumor progression may benefit from preoperative chemotherapy and lymphadenectomy [1, 8–10].

An updated meta-analysis of studies on the prognostic value of preoperative blood-based biomarkers in patients with UTUC treated by RNU showed that several preoperative laboratory abnormalities were associated with an increased risk of cancer-specific mortality [11]. We have previously reported the prognostic value of the De Ritis ratio and the albumin-to-globulin ratio in a large multi-institutional cohort; however, these ratios did not retain their independent prognostic value in multivariate analyses adjusted for standard clinicopathologic features [12].

It has been reported that inflammation has a significant effect on the cancer microenvironment and supports tumor progression [13]. The systemic immune–inflammation index (SII), a novel immune and inflammatory index based on neutrophil, lymphocyte, and platelet counts, has been shown to be associated with oncological outcomes in several types of cancer [14, 15]. However, the value of the SII as a predictor of oncological outcomes in patients with UTUC treated by RNU remains unclear with only two single-center studies in Asia reporting an association between the SII and UTUC in a limited number of subjects [16, 17]. Therefore, the aim of the present study was to externally validate the prognostic significance of the preoperative SII in a large multi-institutional cohort from the international UTUC collaboration.

## Materials and methods

### Patient selection

This study obtained institutional review board approval at each participating institution in the USA and Europe, with all these sites agreeing on institutional data sharing prior to study initiation. This study included a total of 2492 patients treated with open RNU for clinically non-distant metastatic UTUC (Ta-T4N0-1M0) at institutions participating in the UTUC Collaboration between 1990 and 2008. All patients were histologically confirmed to have urothelial carcinoma with only minor involvement of variant components, if any. Patients with missing data or a follow-up < 3 months (*n* = 119) were excluded. No patient received neoadjuvant chemotherapy (NAC) or radiotherapy.

## Data collection and pathologic evaluation

Pretreatment SII values were assessed within 30 days prior to RNU. SII was calculated as platelet count × neutrophil/lymphocyte count. SII and demographic, surgical, pathological, and survival outcomes data were collected and entered into a computerized database. The optimal SII cutoff value was defined by creating a time-dependent receiver operating characteristic (ROC) curve with CSS as the endpoint to yield the highest Youden index value. Briefly, the Youden index provides the optimal cutoff from a continuous variable by showing the score that offers the best trade-off between sensitivity and specificity. Using this score, the overall population was divided into two separate SII groups (> 485 vs. ≤ 485). The 2002 American Joint Committee on Cancer—Union International Centre le Cancer Tumor–Node–Metastasis classification and the 1998 WHO/International Society of Urologic Pathology consensus classification were used for pathologic staging and grading, respectively. The tumor location was categorized as renal pelvicalyceal or ureteral [4], and the predominant tumor architecture pattern was categorized as papillary or sessile [18]. Coexistence of two or more pathologically confirmed urothelial cancers in any location within the upper urinary tract was considered as multifocal disease [19]. Lymphovascular invasion was judged to be present when cancer cells were found within an endothelium-lined space without an underlying muscle wall [20]. Muscle-invasive disease was defined as ≥ pT2 and non-organ-confined (NOC) disease as ≥ pT3 and/or lymph node-positive disease.

## Management and follow‑up

All patients underwent a standard RNU with bladder cuff removal with curative intent. Regional lymphadenectomy and/or adjuvant chemotherapy was performed at the discretion of the urologist. All patients were followed up according to the relevant institutional protocols in accordance with local guidelines at the time. Generally, patients were seen at quarterly intervals postoperatively for the first year, 6-monthly in the second year, and annually thereafter. Follow-up visits consisted of a physical examination, serum chemistry evaluation, urinary cytology, and endoscopic examination of the bladder. Chest radiography and diagnostic imaging of the contralateral upper urinary tract, with a CT urogram, ultrasonography, and/or an intravenous pyelogram, were performed annually. Chest CT and a bone scan were performed at the discretion of the physicians. Recurrences in the bladder or contralateral upper urinary tract were considered as second primaries. Outcomes were measured by time to disease recurrence or to cancer-specific death. The cause of death was determined by the treating physician based on chart review corroborated by the death certificates or by the death certificates alone.

## Statistical analysis

Associations of the SII with categorical variables were assessed using chi-square tests and differences in continuous variables were analyzed using Mann–Whitney *U* tests. Recurrence-free survival (RFS), cancer-specific survival (CSS), and overall survival (OS) were graphically visualized using the Kaplan–Meier method. Difference between groups was assessed with the log-rank test. Univariable and multivariable logistic regression analyses were performed to investigate the association of SII with muscle-invasive and NOC disease. The area under the ROC curve was calculated to determine the discrimination of the logistic regression models. DeLong’s test was used to test for statistical significance between different areas under the curve. Univariable and multivariable Cox regression models were used to investigate the associations of SII with RFS, CSS, and OS. The discrimination of the model was evaluated using the Harrell’s concordance index. The additional clinical net benefit of SII was evaluated using decision curve analysis (DCA). A sub-analysis of survival by tumor location (ureter tumor vs. renal tumor) was also implemented. All *P* values were two-sided, and statistical significance was defined as *P* < 0.05. Statistical analyses were performed using R version 3.6.3 (R Foundation for Statistical Computing, Vienna, Austria) and Stata/MP 14.2 statistical software (Stata Corp., College Station, TX, USA).

## Results

### Patient demographics and their association with the SII

The “best cutoff value” was calculated using the Youden index and identified as 485. Table 1 summarizes the clinicopathologic characteristics of the study cohort. Regional lymphadenectomy was performed in 776 patients (32.7%), and adjuvant chemotherapy was administered in 242 (10.2%). SII > 485 was observed in 986 patients (41.6%) and was associated with a more advanced pathological tumor stage (*P* = 0.03; Table 1). In contrast, there was no significant difference between SII > 485 and SII ≦ 485 in regard to other high-risk pathological features. Moreover, SII > 485 was significantly associated with ureter tumor (*P* < 0.001).

**Table 1:** Association of SII and clinicopathologic characteristics in 2373 patients treated with radical nephroureterectomy for upper tract urothelial carcinoma

	All	SII ≦ 485	SII > 485	*P*
Total, *n* (%)	2373	1387 (58.4%)	986 (41.6%)	
Age, Median	69	69	69	0.72
Female gender, *n* (%)	776 (32.7%)	460 (33.2%)	316 (32.0%)	0.60
Tumor stage, *n* (%)				**0.03**
pTa	501 (21.1%)	298 (21.5%)	203 (20.6%)	
pTis	48 (2.0%)	30 (2.2%)	18 (1.8%)	
pT1	539 (22.7%)	342 (24.7%)	197 (20.0%)	
pT2	453 (19.1%)	254 (18.3%)	199 (20.2%)	
pT3	723 (30.5%)	410 (29.6%)	313 (31.7%)	
pT4	109 (4.6%)	53 (3.8%)	56 (5.7%)	
High grade, *n* (%)	2002 (92.1%)	1168 (84.2%)	834 (84.6%)	0.85
Lymph node status, *n* (%)				0.28
pNx	1597 (67.3%)	943 (68.0%)	654 (66.3%)	
pN0	564 (23.8%)	331 (23.9%)	233 (23.6%)	
pN1	212 (8.9%)	113 (8.1%)	99 (10.0%)	
Lymphovascular invasion, *n* (%)	553 (23.3%)	307 (22.1%)	246 (24.9%)	0.12
Concomitant carcinoma in situ, *n* (%)	558 (23.5%)	332 (23.9%)	226 (22.9%)	0.60
Multifocality, *n* (%)	559 (23.6%)	322 (23.2%)	237 (24.0%)	0.68
Necrosis, *n* (%)	552 (23.3%)	310 (22.4%)	242 (24.5%)	0.23
Architecture, *n* (%)				0.10
Papillary	1796 (75.7%)	1067 (76.9%)	729 (73.9%)	
Sessile	577 (24.3%)	320 (23.1%)	257 (26.1%)	
Location				** < 0.001**
Renal	1526 (64.3%)	1065 (76.8%)	461 (46.8%)	
Ureter	847 (35.7%)	322 (23.2%)	525 (53.2%)	
Bladder carcinoma history, *n* (%)	626 (26.4%)	351 (25.3%)	275 (27.9%)	0.17
Adjuvant chemotherapy, *n* (%)	242 (10.2%)	134 (9.7%)	108 (11.0%)	0.34

Bold *P* values are considered statistically significant (*P* value < .05)

SII, systemic immune–inflammation index

## Association of the risk group with the SII

In univariable logistic regression analyses, SII was significantly associated with muscle-invasive (odds ratio [OR] 1.27, 95% confidence interval [CI] 1.08–1.50, *P* = 0.004) and NOC disease (OR 1.21, 95% CI 1.02–1.43, *P* = 0.03) (Table 2). On multivariable logistic regression analyses adjusted for patients’ age, sex, tumor location, tumor architecture, and history of bladder carcinoma, SII remained independently associated with muscle-invasive disease (OR 1.25, 95% CI 1.05–1.50, *P* = 0.01) but not with NOC. Addition of SII to the multivariable models for the association of NOC and muscle-invasive disease marginally improved their discrimination (Table 2) of the models for predicting NOC disease (accuracy, 73%; *P* = 0.14) by 1 point and for predicting muscle-invasive disease (accuracy, 70%; *P* = 0.02) by 2 points. On DCA, the inclusion of SII did not improve the net benefit of the models across any threshold probability (Supplementary Fig. 1).

**Table 2:** Logistic regression preoperative model including SII for predicting NOCD and MID

	NOCD				MID			
	Univariable		Multivariable		Univariable		Multivariable	
	OR (95%CI)	*P*	OR (95%CI)	*P*	OR (95%CI)	*P*	OR (95%CI)	*P*
Age	1.01 (1.00–1.01)	0.15	1.00 (1.00–1.01)	0.33	1.01 (1.00–1.01)	0.17	1.00 (0.99–1.01)	0.47
Gender	1.06 (0.89–1.27)	0.51	0.94 (0.77–1.15)	0.55	1.07 (0.90–1.27)	0.44	0.99 (0.81–1.19)	0.88
Location	0.64 (0.53–0.76)	** < 0.001**	0.53 (0.43–0.64)	** < 0.001**	0.86 (0.73–1.02)	0.09	0.83 (0.69–1.00)	0.05
BCa history	0.71 (0.59–0.86)	** < 0.001**	0.72 (0.58–0.90)	**0.003**	0.77 (0.64–0.92)	**0.005**	0.75 (0.62–0.93)	**0.007**
Architecture	7.05 (5.72–8.68)	** < 0.001**	7.51 (6.06–9.31)	** < 0.001**	8.81 (6.79–11.45)	** < 0.001**	8.88 (6.83–11.54)	** < 0.001**
SII	1.21 (1.02–1.43)	**0.03**	1.19 (0.99–1.44)	0.06	1.27 (1.08–1.50)	**0.004**	1.25 (1.05–1.50)	**0.01**
Accuracy with SII	0.73				0.70			
Accuracy without SII	0.72				0.68			

Bold *P* values are considered statistically significant (*P* value < .05)

BCa, bladder carcinoma; CI, confidence interval; MID, muscle-invasive disease; NOCD, non-organ-confined disease; OR, odds ratio; SII, systemic immune–inflammation index

**Fig. 1:**
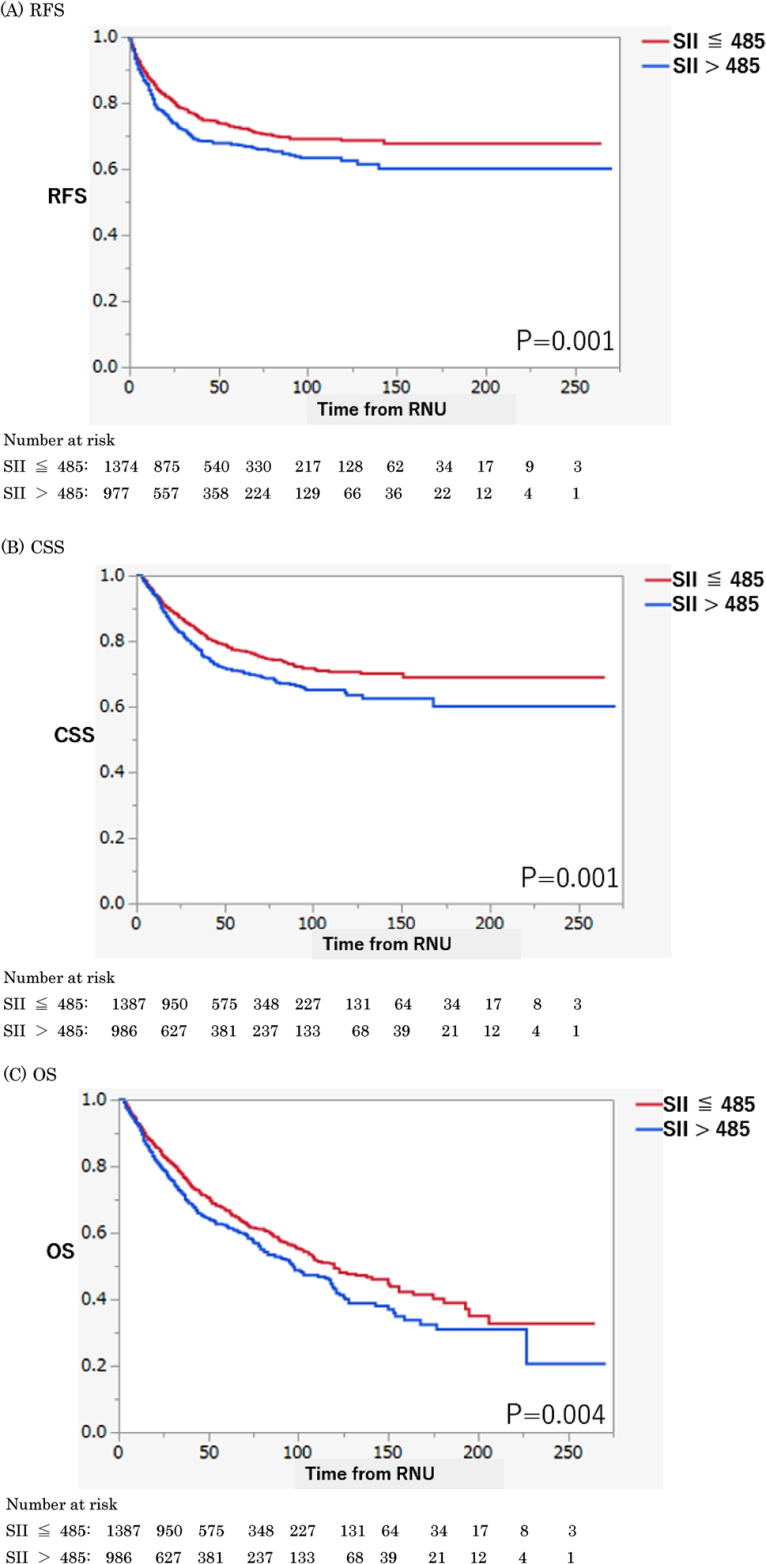
Kaplan–Meier estimates of oncological outcomes stratified by systemic immune–inflammation index (SII) in 2373 patients with upper tract urothelial carcinoma treated with radical nephroureterectomy (RNU). **a** Recurrence-free survival. **b** Cancer-specific survival. **c** Overall survival

## Association of the SII with recurrence and survival

Within a median follow-up of 38 months, 643 patients (27.1%) experienced disease recurrence and 533 patients (22.5%) died from their cancer. Patients with SII > 485 had worse RFS, CSS, and OS than those with an SII ≦ 485 in the respective log-rank test (*P* = 0.001; *P* = 0.001; *P* = 0.004; Fig. 1). On Cox regression analyses, SII > 485 was associated with shorter CSS (hazard ratio [HR] 1.32, 95% CI 1.11–1.56, *P* = 0.002), OS (HR 1.22, 95% CI 1.07–1.40, *P* = 0.004), and RFS (HR 1.29, 95% CI 1.10–1.50, *P* = 0.002) (Table 3). On multivariable Cox regression analyses adjusted for established clinicopathologic features, SII remained independently associated survival outcomes (all *P* < 0.05; Table 3). Addition of the SII to the multivariable models did not improve their discrimination. On DCA, inclusion of the SII did not improve the net benefit of the models across any threshold probability (Supplementary Figure 2).

**Table 3:** Cox regression analyses predicting survival outcomes

	CSS				OS				RFS			
	Univariable		Multivariable		Univariable		Multivariable		Univariable		Multivariable	
	HR (95% CI)	*P*	HR (95% CI)	*P*	HR (95% CI)	*P*	HR (95% CI)	*P*	HR (95% CI)	*P*	HR (95% CI)	*P*
Age	1.02 (1.01–1.03)	** < 0.001**	1.02 (1.01–1.03)	** < 0.001**	1.04 (1.03–1.05)	** < 0.001**	1.04 (1.03–1.05)	** < 0.001**	1.01 (1.01–1.02)	** < 0.001**	1.01 (1.01–1.02)	** < 0.001**
Gender	1.03 (0.86–1.23)	0.74	0.95 (0.79- 1.14)	0.6	1.08 (0.94–1.25)	0.26	0.96 (0.83–1.11)	0.55	1.07 (0.90–1.25)	0.44	1.04 (0.88–1.22)	0.67
Location	1.05 (0.88–1.25)	0.6	1.13 (0.94–1.36)	0.19	1.06 (0.92–1.22)	0.42	1.10 (0.95–1.27)	0.21	1.01 (0.86–1.19)	0.9	1.07 (0.90–1.26)	0.44
Multi	1.28 (1.05–1.54)	**0.01**	0.97 (0.79–1.18)	0.75	1.19 (1.02–1.39)	**0.03**	0.99 (0.84–1.16)	0.89	1.25 (1.05–1.49)	**0.014**	0.91 (0.76–1.09)	0.3
Necrosis	2.24 (1.87–2.67)	** < 0.001**	1.05 (0.87–1.27)	0.58	1.91 (1.65–2.21)	** < 0.001**	1.14 (0.97–1.33)	0.11	2.18 (1.85–2.56)	** < 0.001**	1.06 (0.89–1.26)	0.49
LVI	3.50 (2.94–4.15)	** < 0.001**	1.51 (1.25–1.82)	** < 0.001**	2.46 (2.13–2.83)	** < 0.001**	1.37 (1.17–1.61)	** < 0.001**	3.29 (2.81–3.84)	** < 0.001**	1.42 (1.19–1.69)	** < 0.001**
Grade	7.51 (4.73–12.89)	** < 0.001**	1.97 (1.13–3.43)	**0.02**	2.44 (1.94–3.10)	** < 0.001**	1.21 (0.92–1.59)	0.16	6.45 (4.33–10.18)	** < 0.001**	1.95 (1.22–3.13)	**0.005**
Stage		** < 0.001**		** < 0.001**		** < 0.001**		** < 0.001**		** < 0.001**		** < 0.001**
Reference: pTa, Tis, T1												
pT2	3.53 (2.63–4.77)	** < 0.001**	2.44 (1.77–3.38)	** < 0.001**	1.78 (1.46–2.16)	** < 0.001**	1.48 (1.19–1.84)	**0.03**	3.05 (2.35–3.97)	** < 0.001**	2.13 (1.61–2.83)	** < 0.001**
pT3, T4	9.21 (7.22–11.89)	** < 0.001**	4.89 (3.62–6.60)	** < 0.001**	3.74 (3.20–4.38)	** < 0.001**	2.56 (2.10–3.14)	** < 0.001**	7.59 (6.15–9.45)	** < 0.001**	3.86 (2.98–5.01)	** < 0.001**
Architecture	3.53 (2.97–4.19)	** < 0.001**	1.54 (1.27–1.87)	** < 0.001**	2.57 (2.22–2.96)	** < 0.001**	1.39 (1.18–1.63)	** < 0.001**	3.43 (2.93–4.01)	** < 0.001**	1.61 (1.35–1.91)	** < 0.001**
CIS	1.55 (1.28–1.86)	** < 0.001**	1.03 (0.84–1.26)	0.77	1.39 (1.19–1.61)	** < 0.001**	1.08 (0.91–1.27)	0.37	1.63 (1.38–1.93)	** < 0.001**	1.11 (0.92–1.33)	0.26
AC	3.49 (2.84–4.26)	** < 0.001**	1.71 (1.38–2.13)	** < 0.001**	2.30 (1.90–2.77)	** < 0.001**	1.41 (1.16–1.73)	** < 0.001**	3.97 (3.30–4.75)	** < 0.001**	2.02 (1.65–2.46)	** < 0.001**
SII	1.32 (1.11–1.56)	**0.002**	1.21 (1.02–1.43)	**0.03**	1.22 (1.07–1.40)	**0.004**	1.18 (1.03–1.35)	**0.02**	1.29 (1.10–1.50)	**0.002**	1.18 (1.01–1.37)	**0.04**
C index with SII	0.80				0.74				0.78			
C index without SII	0.79				0.74				0.78			

Bold *P* values are considered statistically significant (*P* value < .05)

CI, confidence interval; CIS, carcinoma in situ; CSS, cancer-specific survival; HR, hazard ratio; LVI, lymphovascular invasion; Multi, multifocality; OS, overall survival; RFS, recurrence-free survival; SII, systemic immune–inflammation index

## Association of the SII with recurrence and survival (ureter tumor vs renal tumor)

Ureter tumor patients with SII > 485 had worse RFS, CSS, and OS than those with SII ≤ 485 in the respective log-rank test (*P* = 0.005; *P* = 0.005; *P* = 0.01; Supplementary Fig. 3). On multivariable Cox regression analyses adjusted for established clinicopathologic features, SII remained independently associated with all survival outcomes (all *P* < 0.05; Supplementary Table 1). In contrast, on multivariable Cox regression analyses, SII failed to show independent prognostic value for all survival outcomes in patients with renal tumor (all *P* > 0.05; Supplementary Table 1). Addition of the SII to the multivariable models did not improve their discrimination in patients with ureter tumor.

## Discussion

In this study, we assessed the significance of the preoperative SII in a large multi-institutional cohort of patients with UTUC treated by RNU. We demonstrated that an altered SII value was not only associated with worse oncological outcomes but predicted the presence of muscle-invasive disease. However, the preoperative SII does not improve net benefit on DCA for either pathologic or survival outcomes. Thus, despite its potential prognostic value, the SII appears to have relatively limited incremental additive value for preoperative clinical decision-making.

We demonstrated that the SII value was associated with oncological outcomes, including the RFS, CSS, and OS. After adjustment for standard pathological prognostic factors in UTUC, preoperative SII retained its statistical significance. These findings are consistent with two previous reports in Asia on the prognostic value of the SII for survival outcomes in UTUC [16, 17]. One strength of the current study is that, unlike two earlier publications, which involved smaller population samples and reflected the single-institution experiences, this study reflected the experience of multiple large international institutions, thus rendering these results more generalizable. In addition, this study included not only survival but also pathological surgical outcomes and thus is more relevant to clinical practice than the most recent study by Zheng et al. [17]. Moreover, the two earlier studies focused on the prognostic value of SII combined with some other parameter but not that of SII alone. Thus, this study may be of value, in that it provides insight into the real preoperative prognostic value of SII.

The mechanisms responsible for the clinical significance of this marker with respect to survival might be explained by the functioning of neutrophils, platelets, and lymphocytes. Neutrophils secrete cytokines and chemokines, such as vascular endothelial growth factor, that enhance tumor angiogenesis, promote adhesion of circulating tumor cells, and facilitate distant metastasis [21–23]. Platelets act to protect cancer cells from immune cell cytotoxicity and facilitate extravasation of cancer cells, leading to formation of a metastatic niche and contributing to the survival and metastasis of cancer cells [24–26]. Platelets may also stimulate tumor cells directly, thereby promoting tumor growth, invasion, and angiogenesis [27]. In contrast, following antigen stimulation, lymphocytes generate important cellular components of the immune response and secrete cytokines (e.g., interferon-γ and tumor necrosis factor-α) that trigger specific immune responses, thereby controlling tumor growth and improving the prognosis in patients with cancer [28–30]. Therefore, immune reactions may be inadequate in patients with cancer and low lymphocyte counts. In light of these mechanisms, a higher SII combined with an increased neutrophil or platelet count or a decreased lymphocyte count is assumed to enhance tumor angiogenesis, adhesion, and metastasis and lead to poor immune clearance of cancer cells.

Moreover, it has been reported that inflammation significantly affects the tumor microenvironment, thereby favoring tumor progression [13]. Cancer and inflammation are linked by both extrinsic and intrinsic pathways, with the former activated by infection or chronic inflammation and the latter driven by genetic changes, such as activation of oncogenes or deactivation of tumor suppressor genes. Both pathways activate key transcription factors in tumor cells, primarily nuclear factor-kB, signal transducer and activator of transcription 3, and hypoxia-inducible factor 1a. In turn, inflammatory mediators and cyclooxygenase-2 are produced, leading to cancer-related inflammation and further promotion of tumor progression [31]. Therefore, elevation of biomarkers of the systemic inflammatory response impacts the growth and progression of cancer [32–34].

In our preoperative model, which included patient age, sex, tumor location, architecture, and history of bladder cancer, we found that the SII independently predicted muscle-invasive disease. Previous models that can be adapted for clinical decision-making have been proposed to identify these patients [3, 5, 35]. Margulis et al. developed a model to predict muscle-invasive disease using preoperative clinicopathologic features, including age, sex, tumor location, architecture, and grade on biopsy [3]. Another predictive model combined high grade, tumor location, local invasion, and hydronephrosis on imaging and was found to have an accuracy of 71% for predicting muscle-invasive disease and 70% accuracy for predicting NOC disease [5]. We constructed a model that included the SII but not grade at biopsy, tumor size, or tumor invasiveness as assessed by preoperative imaging. Therefore, our analysis relied only on easily accessible and reproducible factors for prediction of NOC and muscle-invasive disease. As a result, our model was found to have 70% accuracy for predicting muscle-invasive disease and 73% accuracy for predicting NOC disease, thus achieving superior accuracy to that reported in earlier preoperative models.

Given that the SII values varied depending on the tumor location, we implemented further analyses and demonstrated that the SII was associated with unfavorable survival outcomes in ureter tumors, thus hinting at differences in tumor behavior between renal and ureter UTUC, of which the SII is only one aspect. In this cohort, ureter and renal tumors also differed in tumor stage, lymph node stage, multifocality, necrosis, and concomitant carcinoma in situ. These differences may be accounted for by differences in symptom development leading to diagnosis or tumor biology as follows: that ureter tumors become symptomatic earlier by causing obstruction at earlier stages and grades and thus become detectable by endoscopy earlier, while renal tumors may progress before they become symptomatic or lead to obstruction [36, 37]]. As a result, ureter tumors tend to be detected earlier than renal tumors; thus, there is a possibility of relatively accentuating the importance of the SII for pathological findings in ureter tumors. Nonetheless, addition of the SII to the multivariable models did not improve their discrimination in patients with ureter tumor and it remains unclear why ureter tumors are associated with high SII values. Further study is required to evaluate the prognostic value of the SII by UTUC site, which has never been addressed.

Despite being the largest to investigate the prognostic value of the pretreatment SII in terms of outcomes after RNU, this study has some limitations. First, its retrospective and multicenter design may have resulted in variations in the laboratory, pathological, and surgical workup that could have confounded the results. The SII was determined preoperatively with a predefined cutoff value and analyzed as a categorical variable; thus, different cutoff values may have led to different conclusions. In addition, despite being similar to that used in the two earlier studies, the approach used to determine the cutoff value in this study led to varying cutoff values being used among the studies. Thus, the best SII cutoff value remains unclear. A further major limitation of the study is that the SII was only evaluated as a categorical variable, and not as a continuous variable, thus possibly reducing the predictive ability of the SII as an independent variable. Thus, the SII requires to be evaluated for its real predictive value in further research. Second, unknown pretreatment factors, such as the presence of minor infections and hematological or immune diseases and use of steroids and other medications, may have affected the SII values, thus leading to systematic bias. Furthermore, the SII value was assessed at a single time point preoperatively. While the pretreatment SII values were uniformly assessed within 30 days prior to RNU, the SII values could have changed in the interval between blood collection and RNU. The SII was not evaluated in this study for its variability over time and in response to therapy or for its relationship to the oncological prognosis of UTUC; therefore, the SII remains to be further tested in future studies. Third, most patients had not undergone regional lymph node dissection. Fourth, prior to RNU, no patients underwent NAC, which may have impacted on the preoperative SII and oncological outcomes. Thus, the effect and predictive value of SII should be evaluated in patients receiving NAC in future studies. Fifth, we did not evaluate the incremental additive value of SII for pathology outcomes in a multivariable logistic regression model incorporating established prognostic variables, such as tumor grade and stage on biopsy, tumor stage and hydronephrosis on imaging, and urine cytology. Thus, it remains unclear whether SII may be shown to have independent value for predicting pathological outcomes in a model incorporating all established factors. Thus, this remains an issue to be addressed in future studies. Finally, the SII was the only focus of this study, while growing evidence suggests that combination with other preoperative markers, such as cellular metabolism, may prove helpful in predicting oncological outcomes in patients with UTUC. Therefore, well-designed prospective studies with prolonged follow-up are required to validate the prognostic value of the SII in this setting and to clarify whether it may help enhance the current tools used for risk stratification of patients with UTUC.

In conclusion, the SII is associated with oncological outcomes in patients with UTUC. Moreover, in the preoperative setting, patients could benefit from its ability to independently predict muscle-invasive disease. However, the preoperative SII does not improve net benefit on DCA for either pathologic or survival outcomes. Thus, despite its potential prognostic value, the SII appears to have relatively limited incremental additive value for preoperative clinical decision-making. Further study of SII in prognosticating UTUC is warranted before routine use in clinical algorithms.

## Supplementary information

Below is the link to the electronic supplementary material.

Supplementary information 1 (PDF 94 kb)

Supplementary information 2 (PDF 213 kb)

Supplementary information 3 (PDF 225 kb)

Supplementary information 4 (PDF 27 kb)

## Abbreviations

CI: Confidence interval
CSS: Cancer-specific survival
CT: Computed tomography
DCA: Decision curve analysis
HR: Hazard ratio
NAC: Neoadjuvant chemotherapy
NOC: Non-organ-confined
OS: Overall survival
RFS: Recurrence-free survival
ROC: Receiver operating characteristic
SII: Systemic immune–inflammation index
UTUC: Upper tract urothelial carcinoma
