# Supplementary material for: Prognostic role of the systemic immune–inflammation index in upper tract urothelial carcinoma treated with radical nephroureterectomy: results from a large multicenter international collaboration
Source: Cancer Immunol Immunother. 2021 Feb 16;70(9):2641–50. doi: 10.1007/s00262-021-02884-w (PMC8360829; doi:10.1007/s00262-021-02884-w)

Supplementary Figure 1

Decision curve analysis for additional net-benefit of preoperative systemic immune-inflammation index after being included to a basic model (consisting of age, gender, location, bladder carcinoma history, and architecture) for prediction of non-organ confined disease and muscle invasive disease

(A) Non-organ confined disease

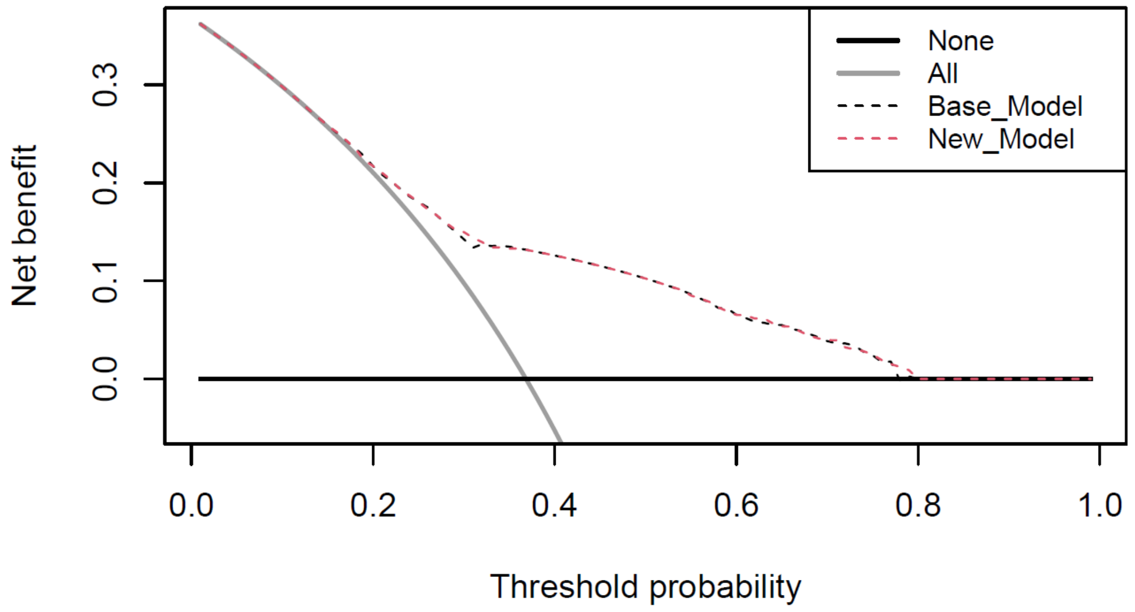

(B) Muscle invasive disease

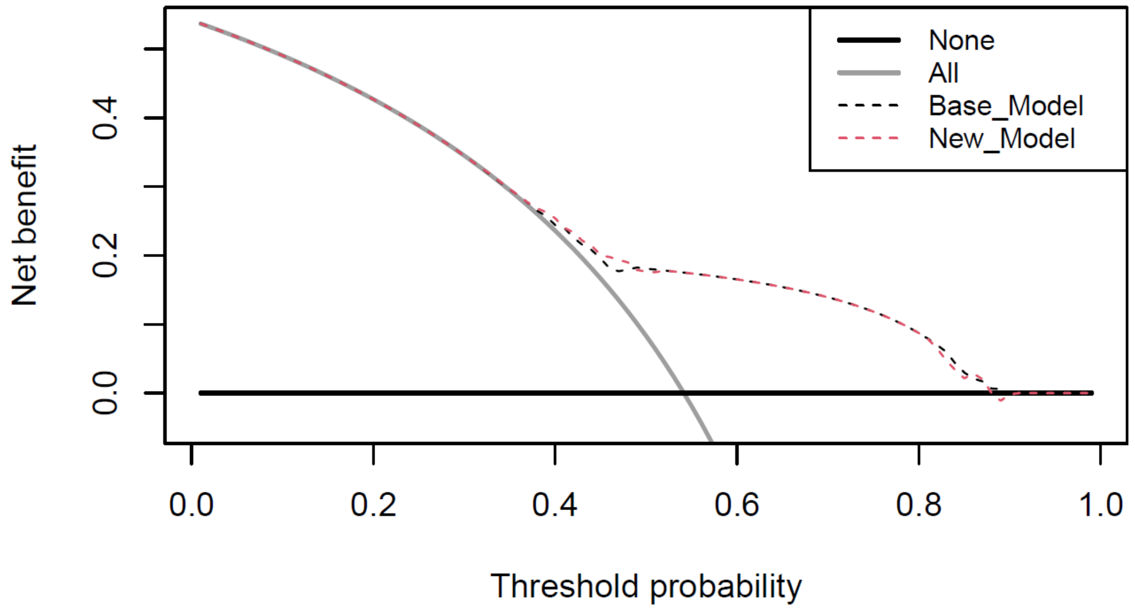

Supplement: Supplementary file 1 — Supplementary information 1 (PDF 94 kb) [file 262_2021_2884_MOESM1_ESM.pdf]
