# Supplementary material for: Prognostic role of the systemic immune–inflammation index in upper tract urothelial carcinoma treated with radical nephroureterectomy: results from a large multicenter international collaboration
Source: Cancer Immunol Immunother. 2021 Feb 16;70(9):2641–50. doi: 10.1007/s00262-021-02884-w (PMC8360829; doi:10.1007/s00262-021-02884-w)

Supplementary Figure 2

Decision curve analyses for additional net-benefit of preoperative systemic immune-inflammation index after being included to a basic model (consisting of established histopathological variables) for prediction of recurrence-free survival, cancer-specific survival and overall survival

(A) Recurrence-free survival

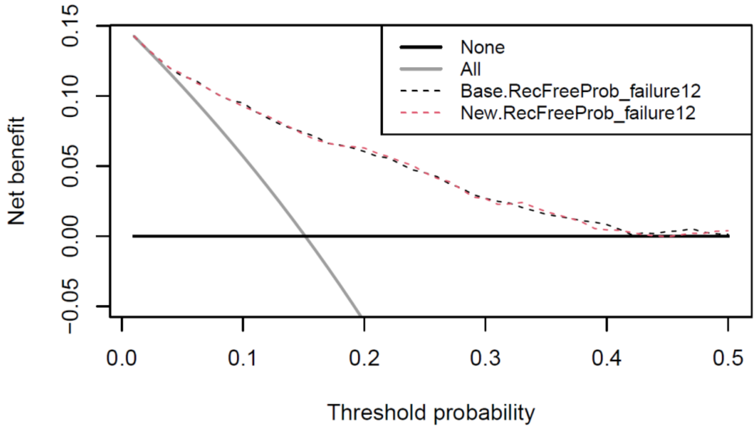

(B) Cancer-specific survival

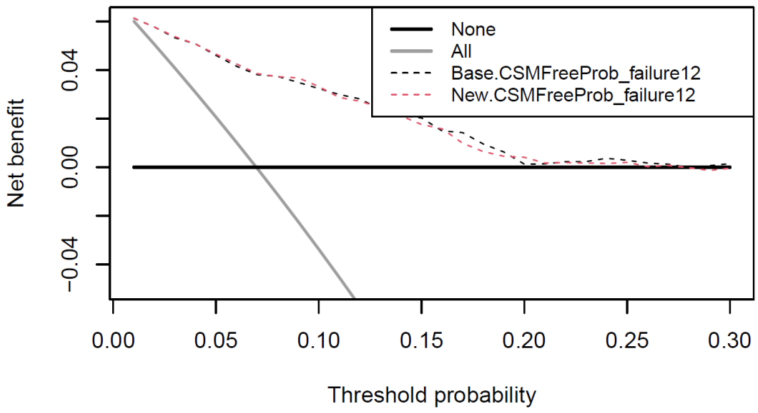

(C) Overall survival

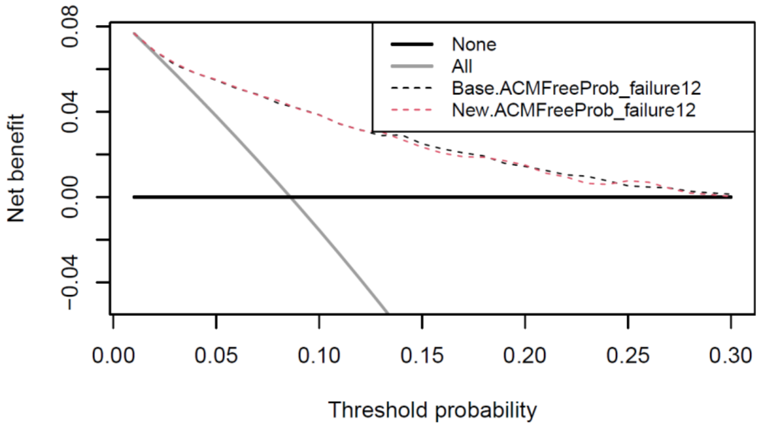

Supplement: Supplementary file 2 — Supplementary information 2 (PDF 213 kb) [file 262_2021_2884_MOESM2_ESM.pdf]
