# Supplementary material for: Prognostic role of the systemic immune–inflammation index in upper tract urothelial carcinoma treated with radical nephroureterectomy: results from a large multicenter international collaboration
Source: Cancer Immunol Immunother. 2021 Feb 16;70(9):2641–50. doi: 10.1007/s00262-021-02884-w (PMC8360829; doi:10.1007/s00262-021-02884-w)

Supplementary Figure 3

Kaplan Meier estimates of oncological outcomes stratified by systemic immune-inflammation index (SII) in patients with upper tract urothelial carcinoma treated with radical nephroureterectomy (RNU)

(A) Recurrence-free survival (RFS) in ureter tumor

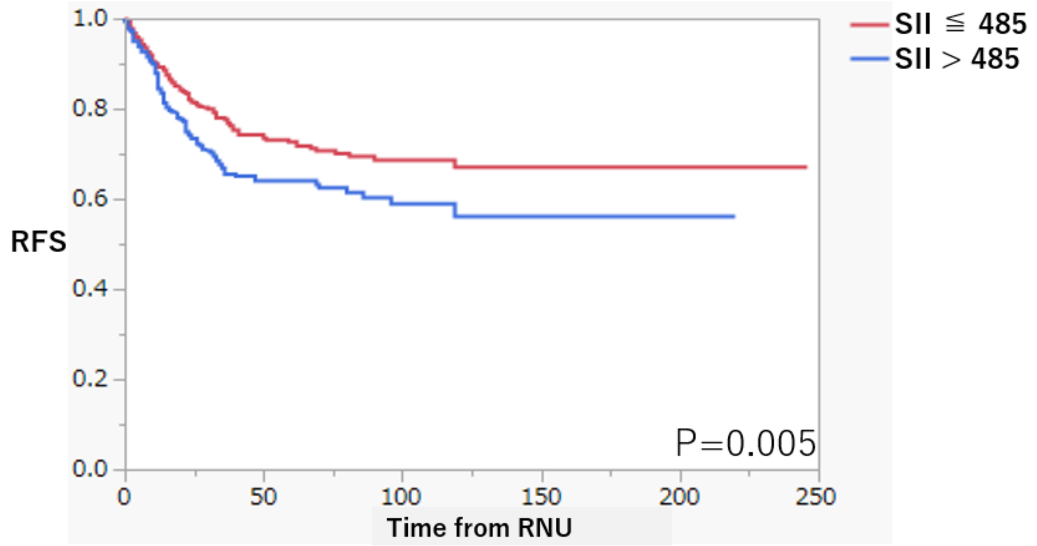

(B) Cancer-specific survival (CSS) in ureter tumor

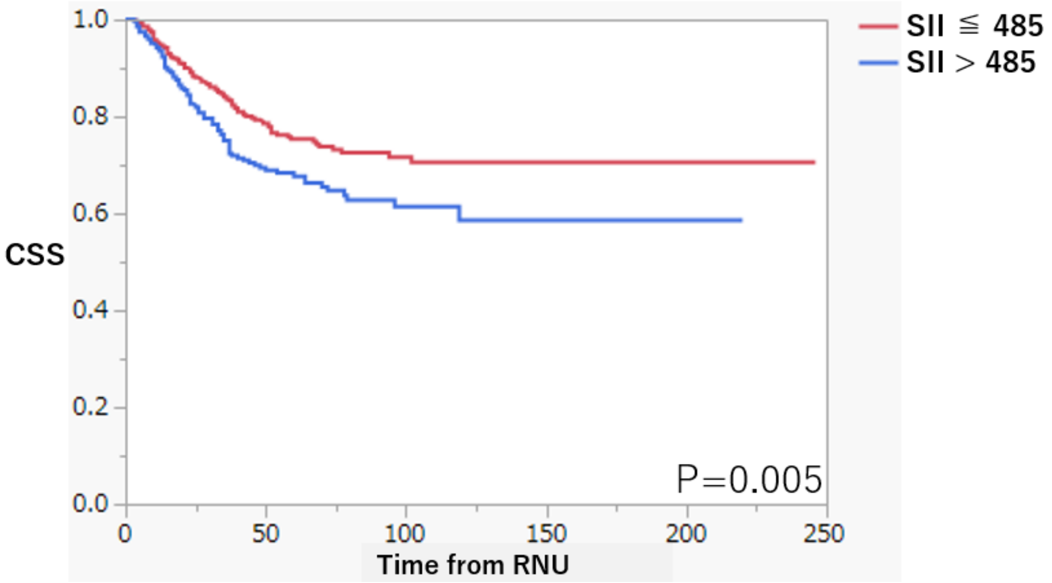

(C) Overall survival (OS) in ureter tumor

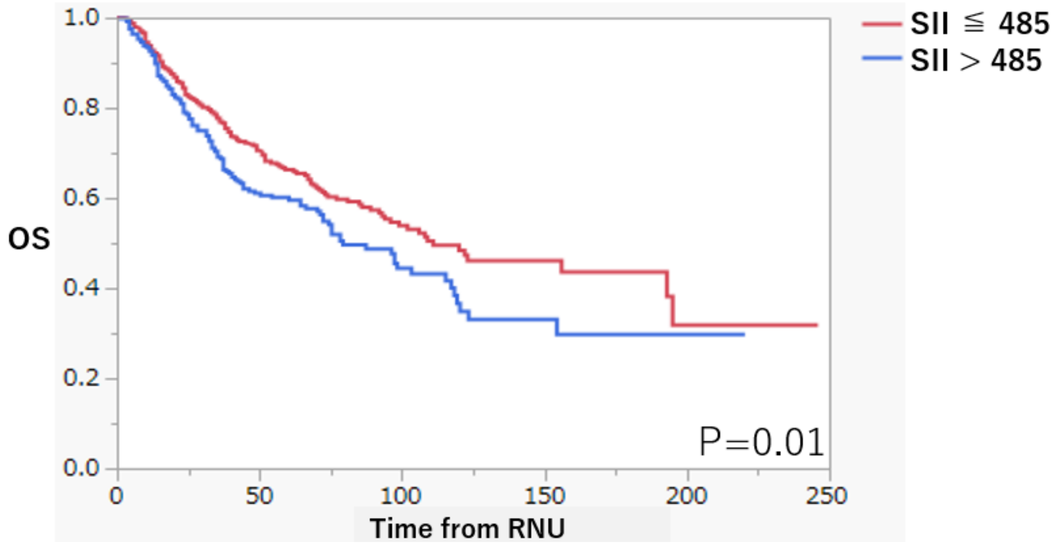

(D) RFS in renal tumor

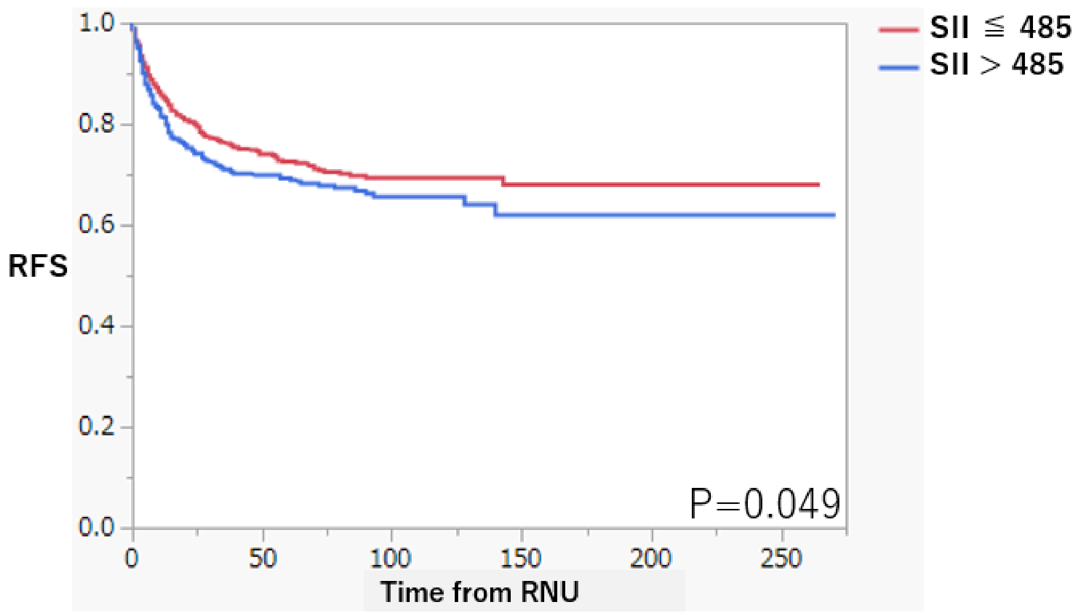

(E) CSS in renal tumor

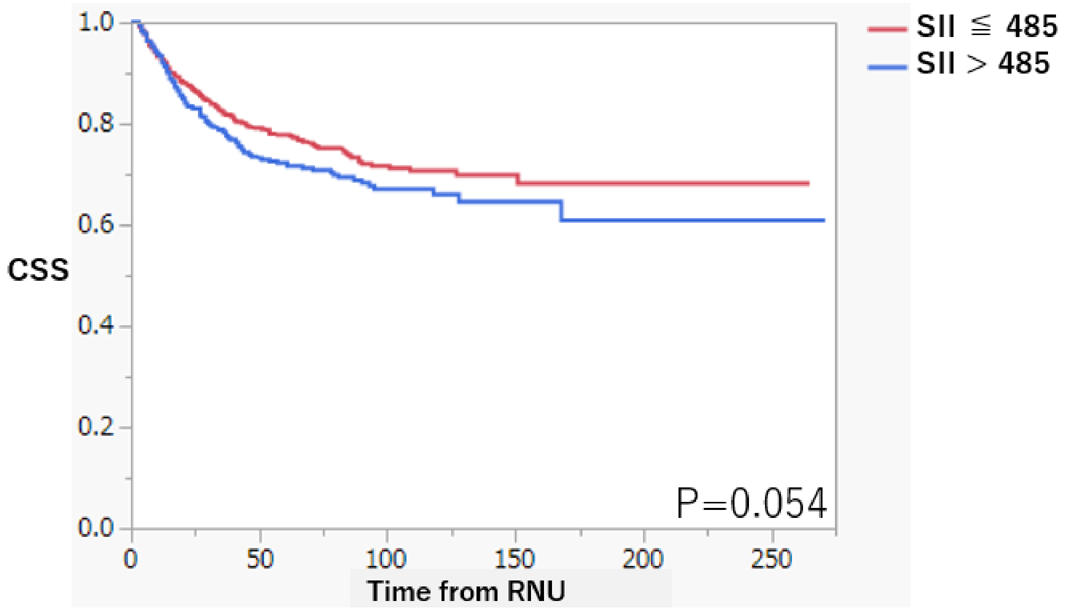

(F) OS in renal tumor

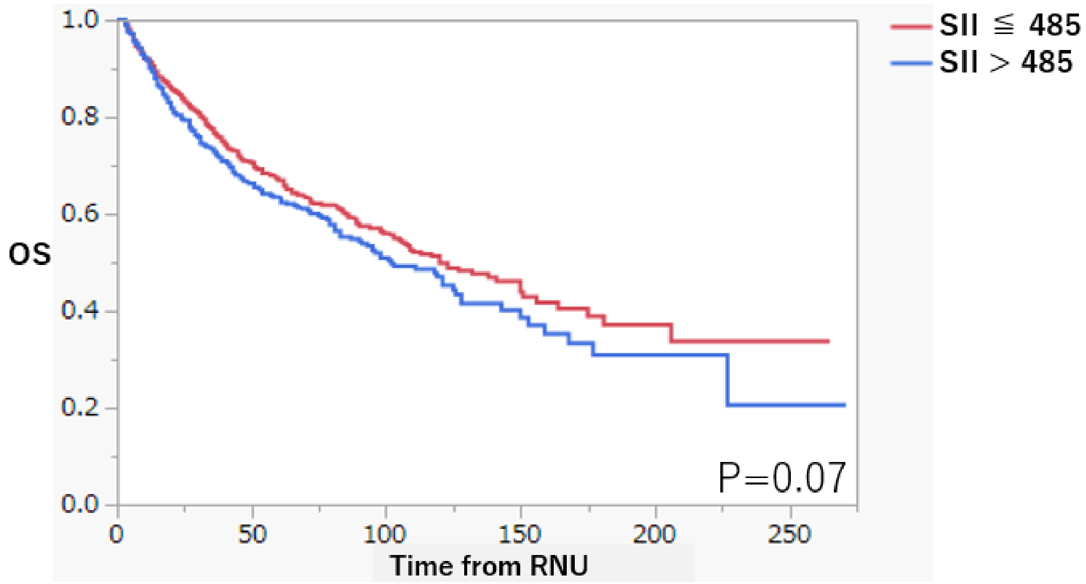

Supplement: Supplementary file 3 — Supplementary information 3 (PDF 225 kb) [file 262_2021_2884_MOESM3_ESM.pdf]
