# Supplementary material for: Prognostic role of the systemic immune–inflammation index in upper tract urothelial carcinoma treated with radical nephroureterectomy: results from a large multicenter international collaboration
Source: Cancer Immunol Immunother. 2021 Feb 16;70(9):2641–50. doi: 10.1007/s00262-021-02884-w (PMC8360829; doi:10.1007/s00262-021-02884-w)
Supplement: Supplementary file 4 — Supplementary information 4 (PDF 27 kb) [file 262_2021_2884_MOESM4_ESM.pdf]

Supplementary Table 1

Cox regression analyses predicting survival outcomes by tumor location (Adjusting age, gender, multifocality, necrosis, LVI, grade, stage, architecture, CIS, and AC)

Ureter tumor						
	CSS		OS		RFS	
	Multivariable		Multivariable		Multivariable	
	HR (95% CI)	*P*	HR (95% CI)	*P*	HR (95% CI)	*P*
SII	1.44 (1.08-1.91)	**0.01**	1.29 (1.03-1.63)	**0.03**	1.37 (1.06-1.78)	**0.02**
C index with SII	0.78		0.73		0.76	
C index without SII	0.77		0.73		0.76	
Renal tumor						
	CSS		OS		RFS	
	Multivariable		Multivariable		Multivariable	
	HR (95% CI)	*P*	HR (95% CI)	*P*	HR (95% CI)	*P*
SII	1.12 (0.90-1.39)	0.31	1.13 (0.96-1.35)	0.15	1.10 (0.90-1.33)	0.36

Bold *P* values are considered statistically significant (*P* value <.05).

Abbreviations: CI = confidence interval; CIS = carcinoma in situ; CSS = cancer-specific survival; HR = hazard ratio; LVI = lymphovascular invasion; OS = overall survival; RFS = recurrence-free survival; SII = systemic immune-inflammation index
